# Intrinsic Brain Activity Responsible for Sex Differences in Shyness and Social Anxiety

**DOI:** 10.3389/fnbeh.2017.00043

**Published:** 2017-03-13

**Authors:** Xun Yang, Ming Zhou, Sunima Lama, Lizhou Chen, Xinyu Hu, Song Wang, Taolin Chen, Yan Shi, Xiaoqi Huang, Qiyong Gong

**Affiliations:** ^1^Huaxi MR Research Center, Department of Radiology, West China Hospital of Sichuan UniversityChengdu, China; ^2^Department of Sociality and Psychology, Southwest University for NationalitiesChengdu, China

**Keywords:** shyness, social anxiety, sex differences, ALFF, fALFF, resting fMRI

## Abstract

Male and female show significant differences in important behavioral features such as shyness, yet the neural substrates of these differences remain poorly understood. Previous neuroimaging studies have demonstrated that both shyness and social anxiety in healthy subjects are associated with increased activation in the fronto-limbic and cognitive control areas. However, it remains unknown whether these brain abnormalities would be shared by different genders. Therefore, in the current study, we used resting-state fMRI (r-fMRI) to investigate sex differences in intrinsic cerebral activity that may contribute to shyness and social anxiety. Sixty subjects (28 males, 32 females) participated in r-fMRI scans, and the amplitude of low-frequency fluctuations (ALFF) and fractional ALFF (fALFF) were used to measure the spontaneous regional cerebral activity in all subjects. We first compared the differences between male and female both in the ALFF and fALFF and then we also examined the whole brain correlation between the ALFF/fALFF and the severity of shyness as well as social anxiety by genders. Referring to shyness measure, we found a significant positive correlation between shyness scores (CBSS) and ALFF/fALFF value in the frontoparietal control network and a negative correlation in the cingulo-insular network in female; while in male, there is no such correlation. For the social anxiety level, we found positive correlations between Leibowitz Social Anxiety Scale (LSAS) scores and spontaneous activity in the frontal-limbic network in male and negative correlation between the frontal-parietal network; however, such correlation was not prominent in female. This pattern suggests that shy female individuals engaged a proactive control process, driven by a positive association with activity in frontoparietal network and negative association in cingulo-insular network, whereas social anxiety males relied more on a reactive control process, driven by a positive correlation of frontal-limbic network and negative correlation of frontoparietal network. Our results reveal that shyness or social anxiety is associated with disrupted spontaneous brain activity patterns and that these patterns are influenced by sex.

## Introduction

Shyness is a fundamental trait that has been conceptualized as anxious self-preoccupation and behavioral inhibition in social contexts that derives from the prospect of interpersonal evaluation (Amico et al., 2004). Findings from a number of longitudinal studies have shown that shyness is a stable and heritable construct that can predict important life outcomes in adulthood, such as interpersonal relations, psychopathology, physical and mental health, occupational attainment, and crime (Shiner et al., 2003).

Investigations into the biological bases of shyness have recently begun and have employed functional imaging techniques to explore possible brain correlates of shyness in healthy individuals. Several task-dependent fMRI studies have found that shy individuals demonstrated increased activation in the frontal cortex and forebrain limbic areas when processing emotional faces (Schwartz et al., 2003; Beaton et al., 2008, 2009, 2010). For example, Schwartz and colleges reported greater amygdalar activation in response to novel faces in young adults who were classified as shy versus non-shy as children (Schwartz et al., 2003). In contrast to task-dependent fMRI studies, resting fMRI studies allow for the investigation of intrinsic or spontaneous brain networks in an ecologically valid manner and avoid some of the constraints of task-dependent paradigms. Using seed-based functional connectivity analysis, we found that shyness is either positively or negatively associated with various brain functional connectivity differences that involve the superior temporal gyrus, parahippocampal gyrus, amygdala, and insula (Yang et al., 2013); these results suggest that these brain areas may constitute a network that is prominently linked to shyness. In a recent investigation, we used functional connectivity strength (FCS), an unbiased method to investigate brain-wide intrinsic connectivity patterns, and observed that the FCS of the insula positively correlated with shyness scores (Yang et al., 2015). This result could indicate impaired neural network communication between the insular hub and other brain regions.

Although past studies have identified how the key node associated with shyness interacts with other connected regions, the regional spontaneous activity in the resting state for shyness remains unknown. The amplitude of low-frequency fluctuations (ALFF) and fractional ALFF (fALFF) are believed to reflect the strength of intrinsic spontaneous neuronal activity. ALFF is defined as the total power within the low-frequency range, typically 0.01–0.1 Hz (Zang et al., 2007), whereas fALFF measures the power within the low-frequency range divided by the total power in the entire detectable frequency range (Zou et al., 2008). Each of these two indicators has its own advantages: ALFF has higher test–retest reliability in gray matter regions than fALFF, whereas fALFF is less susceptible to artifactual contributions of cardiac and respiratory (Zuo et al., 2010). As such, we will use both of these methods in the present analysis. Moreover, the spontaneous fluctuations that occur during the resting state are related to extrinsic behavior performance (Fox and Raichle, 2007). Several previous studies have revealed that spontaneous brain activity is an effective and predictive indicator of personality traits (e.g., Big Five traits, self-esteem, extraversion, and neuroticism) (Kunisato et al., 2011; Wei et al., 2011; Pan et al., 2015), cognitive style (Hao et al., 2013), and emotional intelligence (Pan et al., 2014). However, no studies have investigated the relationship between the amplitude of spontaneous brain activity and shyness. This approach may contribute to the confirmation and elaboration of the biological model of shyness. In the present study, we investigated the relationships between ALFF/fALFF and shyness using resting fMRI.

Gender is an important determinant factor that contributes to individual differences in personality (Arrais et al., 2010). Women report themselves to be higher in neuroticism, agreeableness and more shy and anxious, whereas men report themselves to be higher in assertiveness (Costa et al., 2001). It is postulated that gender creates a huge amount of difference between male and female. In line with this hypothesis are studies that have indicated gender differences for shyness and that have presented higher rates of shyness in females compared to males (Else-Quest et al., 2006; Smith et al., 2012). However, there are also studies demonstrating that the male tends to be shyer than the female (Engfer, 1993) and that the effect of shyness on social behavior may be worse in boys than girls (Howarth et al., 2013). One previous neuroimaging study demonstrated that shyness in healthy subjects is associated with disrupted brain connectivity patterns and that these patterns are influenced by sex: the FCS values of the dorsal anterior cingulate cortex (dACC), insula, and subgenual anterior cingulate cortex positively correlated with shyness scores in females but negatively correlated in males (Yang et al., 2015). Consistent with above findings, Henderson and colleagues reported that shyness was associated with stronger conflict-related ACC activity, as assessed by the N2 ERP component during a modified Flanker task (Henderson, 2010). The regions including dACC and anterior insula are known form the cingulo-insular network, and constitute a functional network of regions co-activating in synchrony both in response to reactive cognitive control, especially in conflict monitoring and adjustment signals (Dosenbach et al., 2007; Seeley et al., 2007). Considering the activation profiles and relevant function in cingulo-insular network, it perhaps reflects enhanced conflict sensitivity in the regulation of attention and emotions associated with shyness. However, the findings of current studies have been somewhat inconsistent. For example, another EEG study on young children demonstrated that shy girls showed greater right mid-frontal activation while seeing emotional clips than shy boys, who displayed greater activation in the left mid-frontal area (Theall-Honey and Schmidt, 2006). As far as we known, the mid-frontal area, especially dorsal lateral frontal area seems to be functionally connected to the inferior parietal gyrus (Corbetta and Shulman, 2002), which belongs to frontoparietal control network, and previously implicated in error-related activity and top–down cognitive control, termed as proactive control. Although the existing links between shyness and control-related brain activity, suggests that shyness may be associated with impairment in aspects of cognitive control, it is still unclear whether different shy individuals with tend to rely on different forms of cognitive control, and whether these control patterns are influenced by sex. Given the inconsistency of the relationship between shyness and gender differences in previous behavior and neuroscience studies, the synchronization of spontaneous BOLD activity study may help to elucidate the biological underpinnings involving regional spontaneous brain mechanisms of the above phenomena.

Social anxiety has been suggested to have a close relationship with shyness and is increasingly recognized as a pervasive problem found in almost all segments of the population (Heiser et al., 2003, 2009). Both of these conditions share many symptoms, including somatic, behavioral, and cognitive symptoms (Heiser et al., 2009). Moreover, shyness and social anxiety are associated with hyper-responsivity to social stimuli in both the frontal cortex and limbic system (Kim et al., 2011; Yang et al., 2013). These shared incidence rates, symptomatology, and brain activity patterns raise the question about the diagnostic boundaries of social anxiety. Although many EEG and fMRI studies have been performed to study shyness or social anxiety separately, to the best of our knowledge, only one study has evaluated them simultaneously (Yang et al., 2013). This study showed that prominent structural and functional connectivity changes are especially associated with levels of shyness rather than social anxiety, despite some behavioral correlations with shyness and social anxiety. However, this study only focused on seed-based functional connectivity between seed regions (structurally changed areas) and other regions and did not consider spontaneous brain activity in shyness and social anxiety or the gender effects for either condition. Interesting, in line with the findings in shyness, studies in social anxiety also found that, compared with participants with lower degree of social anxiety, high social anxiety individuals demonstrated increased activity in cingulo-insular circuit (Schmid et al., 2015). These results suggest that people use different control strategies to enhance their performance depending on their levels of social anxiety. Schmid et al. (2015) proposed the notion that the distinction between frontoparietal network mediated regulative control and cingulo-insular network mediated conflict monitoring can inform the effect of social anxiety on cognitive control, and this may help to explain different self-regulatory impairments in social anxiety. Male and female individuals with social anxiety show significant differences in a number of important behavioral features, yet sex specific or shared the neural substrates underlying social anxiety are still poorly understood.

Thus, in current study we propose that the distinction between these two independent control networks can affect shyness/social anxiety level with cognitive control. We therefore investigate the sex difference-associated altered intrinsic activities in shyness or social anxiety in a cohort of healthy subjects using ALFF and fALFF indexes. We hypothesized that the difference between shyness and social anxiety would be reflected in spontaneous brain activity, especially different cognitive control network, and that this brain activity would be influenced by sex.

## Materials and Methods

### Participants

The study was approved by the local ethics committee of Sichuan University, and each subject provided written informed consent for the study. A total of 61 healthy volunteers (29 males, 32 females, mean age ± SD = 21.96 ± 1.94 years) were recruited and were also the same participants in our previous study (Yang et al., 2013, 2015). Each subject completed the Revised Cheek and Buss Shyness Scale (CBSS) and the Leibowitz Social Anxiety Scale (LSAS) and then received resting fMRI scanning. The sample included 32 individuals (16 males, 16 females) who were approximately in the top and bottom 25% of the CBSS score and 28 individuals (12 males and 16 females) who were in the mid-range of the scale. All of the subjects were right-handed, which was determined using the Edinburgh Handedness Scale, and they were scanned with SCID-NP (Structured Clinical Interview according to DSM-IV None-Patient version) to rule out any current or past history of Axis I diagnosis of psychiatric disorders.

### Behavior Measures

We applied the CBSS and LSAS to measure shyness and social anxiety, respectively, the details of which have been described in our previous study (Yang et al., 2013, 2015). The CBSS Chinese version consists of 13 items designed to assess both the behavioral and subject aspects of shyness. Each item is answered on a 0 (extremely uncharacteristic) to 4 (extremely characteristic) scale and thus produces a total CBSS score ranging from 13 to 65, with higher scores reflecting greater levels of shyness. LSAS is a scale that assesses fear and avoidance in 24 situations that are likely to elicit social anxiety using a 0–3 scale. An overall total score may be derived by summing the fear and avoidance ratings for all 24 items. Both of these two scales have been shown to have high internal consistency and have previously been validated in Chinese subjects (Yang et al., 2013).

### MRI Acquisitions

The resting fMRI images were acquired on a whole-body 3.0T MR scanner (Siemens Trio, Erlangen, Germany) with a 12-channel head coil as the signal receiver. Throughout the resting fMRI scan, the subjects were instructed to relax and to keep their eyes closed and not to fall asleep. The scanning parameters were as follows: TR = 2000 ms; TE = 30 ms; FA = 90; acquisition matrix = 64 × 64; FOV = 240 mm × 240 mm; flip angle = 90°; thickness = 5.0 mm; gap = 0 mm; voxel size = 3.75 mm × 3.75 × 5 mm^3^. Each brain volume comprised 30 axial slices with a total scan time of 414 s.

### Data Processing

Data preprocessing was performed using DPARSF (Data Processing Assistant for Resting-State) software^1^ (Wang et al., 2011). For each participant, the first five images were discarded to ensure steady-state longitudinal magnetization. After slice timing and head motion correction, normalization was conducted, and all of the images were spatially smoothed using a Gaussian kernel of 8 mm full width at half maximum (FWHM). After discarding subjects with excessive head motion that had exceeded ±1.5 mm of displacement or ±1.5° of rotation, we finally managed to obtain 60 subjects in the current study.

Both ALFF and fALFF maps were calculated using REST software^2^. The time series were first transformed to the frequency domain using a fast Fourier transform (FFT), and the power spectrum was then obtained. Because the transformed frequency within the power spectrum is proportional to the square of the amplitude of this frequency component in the original time series, the power spectrum obtained by FFT was calculated and averaged across the frequency range 0.01–0.08 Hz at each voxel over the time courses. This averaged square root was taken as the ALFF value. For the fALFF analysis, the average square root of power in the 0.01–0.08 Hz for each voxel was normalized by the total power across all of the available frequencies for that voxel (Zou et al., 2008), which has been reported to be more sensitive than the original ALFF in detecting spontaneous brain activity (Zou et al., 2008). These images were used for the statistical analysis.

### Statistical Analysis

First, we performed a voxel-based two-sample *t*-test for both the ALFF and fALFF maps between the male and female groups, taking the age and CBSS, and age and LSAS as covariates using SPM8, separately. The regions that showed group differences were extracted for further group comparison between genders with separate low, middle, and high CBSS groups using SPSS. From the distribution of CBSS scores among all subjects, cutoff groups were created based on the quartiles of CBSS score (Heinzer et al., 2015). The upper quartile was defined as the high CBSS group, and the lower quartile as the low group, and those in between the upper and lower quartiles as the middle group.

To determine the effects of sex differences in brain activity related to shyness or social anxiety, we performed a voxel-based multiple regression analysis between shyness or social anxiety in males and females separately, which keep age as a covariate. The threshold of all the imaging statistical results were set at a value of *p* < 0.05 for AlphaSim correction (combined height threshold of *p* < 0.01 and a minimum cluster size of 80 voxels for regression analysis).

## Results

### Characteristics of Subjects

The demographic characteristics of the 60 subjects are summarized in **Table 1**. There were no significant differences in age (*p* = 0.412) or shyness or social anxiety measurements between genders (*p*_-CBSS_ = 0.082, *p*_-LSAS_ = 0.223). CBSS scores were significantly correlated with LSAS scores (*r* = 0.376, *p* = 0.003) for the entire group.

**Table 1:** Demographic data for all of the participants.

Subjects	Male	Female	*p-*value^a^
Gender (M/F)	28	32	–
Age (m ± SD)	22.11 ± 1.93	21.68 ± 1.99	0.412
CBSS	40.29 ± 12.84	34.65 ± 11.79	0.082
LSAS	36.54 ± 18.99	43.75 ± 25.38	0.223
*Total Fear*	17.11 ± 10.59	21.72 ± 13.61	0.153
*Total Avoidance*	19.79 ± 11.83	22.00 ± 12.42	0.484
			
*CBSS low*	23.14 ± 3.68	19.71 ± 3.98	0.120
			
*CBSS middle*	40.69 ± 6.02	32.88 ± 5.31	0.001^∗^
*CBSS high*	54.63 ± 3.68	49.44 ± 6.74	0.064


*^∗^*P-*value < 0.05. ^a^Age and questionnaire scores were compared using independent sample *t*-tests. CBSS, Cheek and Buss Shyness Scale; score ranges from 13 to 65. LSAS, Liebowitz Social Anxiety Scale; score ranges from 0 to 144. This scale includes two important subscales, namely, Total Fear and Total Avoidance, which is derived by summing the fear and avoidance rating for all items. The low, middle and high CBSS subgroup were created based on the quartiles of CBSS score.*

### ALFF/fALFF Comparison of Gender Differences

**FIGURE 1:**
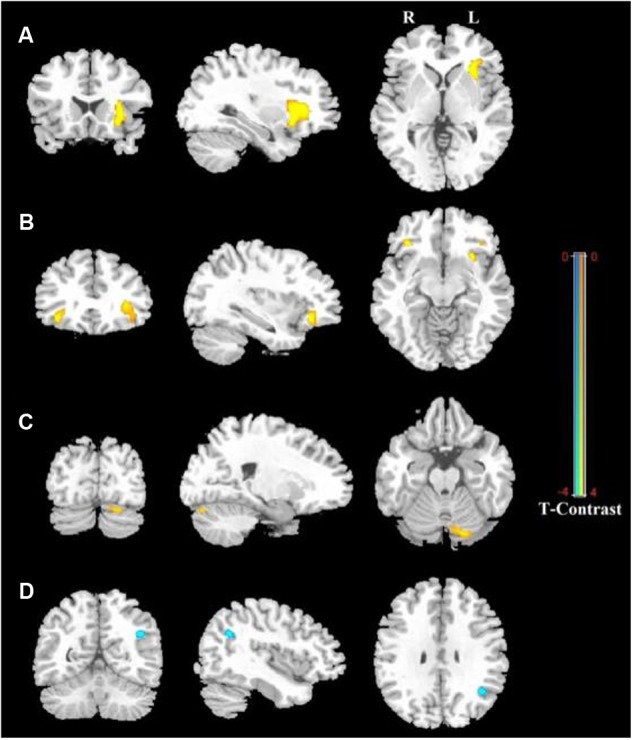
**Regions showing significantly increased and decreased ALFF activity between males and females, with age and CBSS score as covariates.** Yellow color indicates increased activity of the brain in males compared to females, and blue color indicates decreased activity of the brain in females compared to males. **(A)** Left Inferior Frontal Gyrus (LIFG), **(B)** Right Inferior Frontal Gyrus (RIFG), **(C)** Left Cerebellum Posterior Lobe (LCPL), **(D)** Left Inferior Parietal lobule (LIPL). The statistical threshold was set at *p*-value < 0.001, cluster size > 22 mm^3^ (AlphaSim corrected). The figure is shown according to radiological convention.

**FIGURE 2:**
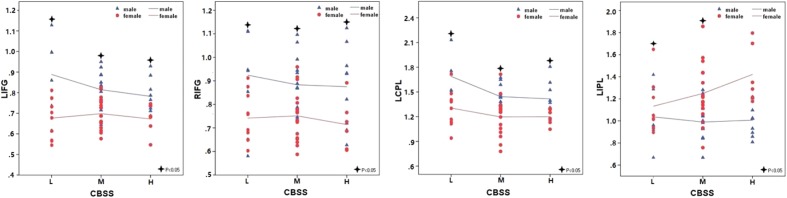
**The comparisons in the high, low, and middle shy subgroup showed significant ALFF differences in LIFG, RIFG and LCPL between males and females and low and middle shy subgroup showed significant ALFF difference in LIPL between males and females.** LIFG, left inferior frontal gyrus; RIFG, right inferior frontal gyrus; LCPL, left cerebellum posterior lobe; LIPL, left inferior parietal lobule; L, low; M, middle; H, high.

**Figure 1** shows the ALFF differences between the male and female groups, taking age and CBSS score as covariates. Compared to the female group, the male group showed increased ALFF levels in some brain regions, most prominently in the bilateral inferior frontal gyrus and the left cerebellum posterior lobe, and decreased activity in the left inferior parietal lobule (*p* < 0.05, AlphaSim correction) (**Table 2**). There was no significant difference between the genders in terms of the fALFF maps. When comparing the male and female groups of ALFF/fALFF with age and LSAS as covariates, we got the similar results (see Supplementary Table S1 and Figure S1). The ROI analysis of the extracted brain regions showed significant ALFF differences between males and females, with significant differences for most comparisons in the high, low, and middle shy subgroups except for the difference in the high shy group in the left inferior parietal lobule (see **Figure 2**).

**Table 2:** Detailed information for voxels showing significant differences between males and females in rs-fMRI ALFF values with age and CBSS score as covariates with AlphaSim correction (cluster size > 22 mm^3^, *p* < 0.001).

Gender	Region	Voxel size	MNI Coordinates X, Y, Z	T value	*P* value (uncorrected)
M > F	L Inferior frontal gyrus	190	-27 18 3	4.97	*p* < 0.001
M > F	R Inferior frontal gyrus	47	36 30 -12	4.37	*p* < 0.001
M > F	L Cerebellum posterior lobe	31	-18 -84 -24	3.98	*p* < 0.001
F > M	L Inferior parietal lobule	25	-39 -60 30	-4.46	*p* < 0.001


*CBSS, Cheek and Buss Shyness Scale; MNI, Montreal Neurological Institute; M, male; F, female; L, Left; R, Right.*

#### Whole Brain Correlation Analysis for Shyness by Gender

**FIGURE 3:**
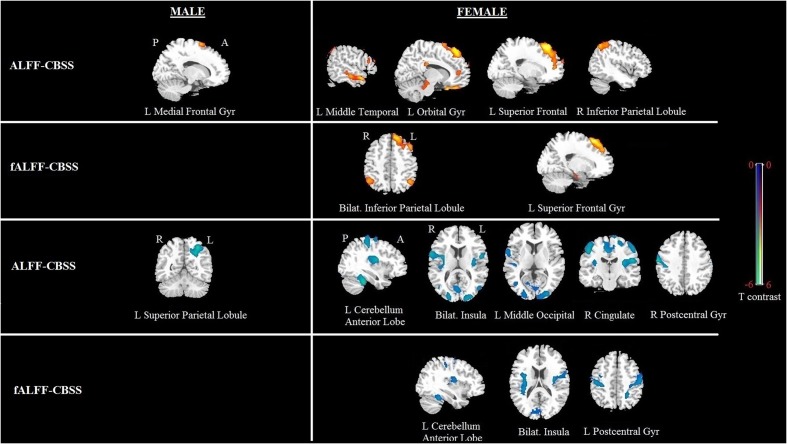
**Brain regions showing a significant correlation between ALFF and fALFF maps and shyness in males and females using voxel-based regression analysis, with age and LSAS as covariates.** Yellow color indicates positive correlation, and blue color indicates negative correlation. The statistical threshold was set at *p*-value < 0.01, cluster size > 80 mm^3^ (with AlphaSim corrected). The figure is shown according to radiological convention.

Significant correlation of shyness with ALFF/fALFF maps in females: A statistically significant positive association between shyness and ALFF maps was found in females in the left middle temporal gyrus, left orbital frontal gyrus, left superior frontal gyrus, and right inferior parietal lobule, whereas a significant negative correlation was found in the left cerebellum anterior lobe, bilateral insula, left middle occipital gyrus, right cingulate gyrus, and right postcentral gyrus. Similar results were found in the correlation analysis between the CBSS and fALFF maps in females; a significant positive correlation was found between the bilateral inferior parietal lobule and right superior frontal gyrus, and a negative correlation in the left cerebellum anterior lobe, bilateral insula, and left postcentral gyrus (see **Figure 3** and **Table 3**).

**Table 3:** Regions showing significant correlation of CBSS scores with ALFF or fALFF maps in males and females separately with AlphaSim correction (*p*-value < 0.01, cluster size > 80 mm^3^).

Brain region	Sex	Voxel size	MNI Coordinates X, Y, Z	*T*-value	*P-*value (uncorrected)
**ALFF-CBSS**					
L Superior frontal gyrus	F	906	-18, 24, 54	4.59	*p* < 0.001
L Orbital gyrus	F	148	-9, 18, -33	4.56	*p* < 0.001
R Inferior parietal lobule	F	174	42, -66, 54	4.13	*p* < 0.001
L Middle temporal gyrus	F	205	-54, 6, -24	3.81	*p* < 0.001
L Insula	F	330	-51, -3, 12	-4.72	*p* < 0.001
L Cerebellum anterior lobe	F	201	-33, -39, -33	-4.52	*p* < 0.001
R Postcentral gyrus	F	1046	63, -12, 36	-4.37	*p* < 0.001
L Middle occipital gyrus	F	311	-27, -93, 6	-3.89	*p* < 0.001
R Insula	F	227	36, -3, 12	-3.82	*p* < 0.001
R Cingualte gyrus	F	118	3, -21, 42	-3.22	0.002
L Medial frontal gyrus	M	121	15, 3, 66	3.8	*p* < 0.001
L Superior parietal lobule	M	253	-30, -57, 51	-4.56	*p* < 0.001
**fALFF-CBSS**					
L Superior frontal gyrus	F	593	-18, 21, 57	4.81	*p* < 0.001
L Superior parietal lobule	F	253	-30, -57, 51	4.56	*p* < 0.001
R Superior parietal lobule	F	253	-30, -57, 51	4.56	*p* < 0.001
R Insula	F	825	36, -3, 18	-5.07	*p* < 0.001
L Cerebellum anterior lobe	F	130	-36, -45, -30	-3.82	*p* < 0.001
L Insula	F	110	-33, -12, 15	-3.74	*p* < 0.001
L Postcentral gyrus	F	252	-51, -18, 48	-3.71	0.001


*CBSS, Cheek and Buss Shyness Scale; MNI, Montreal Neurological Institute; LSAS, Leibowitz Social Anxiety Scale; M, male; F, female.*

Significant correlation of shyness with ALFF/fALFF maps in males: We found a significant positive correlation between the CBSS and ALFF map in the left medial frontal gyrus, and a negative correlation in the left superior parietal lobule. There was no significant correlation between the CBSS and fALFF maps.

### Whole Brain Correlation Analysis for Social Anxiety by Gender

**FIGURE 4:**
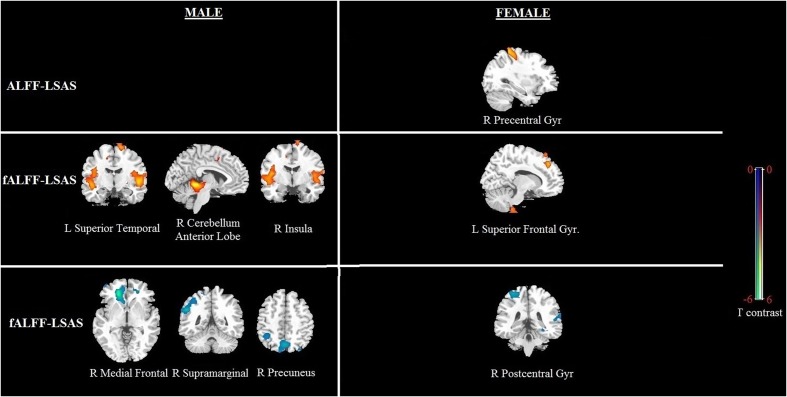
**Brain regions showing a significant correlation between ALFF and fALFF maps and social anxiety in males and females using voxel-based regression analysis, with age and CBSS as covariates.** Yellow color indicates positive correlation, and blue color indicates negative correlation. The statistical threshold was set at *p*-value < 0.01, cluster size > 80 mm^3^ (with AlphaSim corrected). The figure is shown according to radiological convention.

Significant correlation of LSAS with ALFF/fALFF maps in females: For the ALFF maps, the LSAS score showed a positive correlation with the ALFF maps in the right precentral gyrus, and no significant negative correlation was found in females. For the fALFF maps, there was a positive correlation in the left superior frontal gyrus and a negative correlation in the right postcentral gyrus in females (see **Figure 4** and **Table 4**).

**Table 4:** Regions showing significant correlation of LSAS scores with ALFF or fALFF maps in males and females separately with AlphaSim correction (*p*-value < 0.01, cluster size > 80 mm^3^).

Brain region	Sex	Voxel size	MNI Coordinates X, Y, Z	*T*-value	*P-*value
**ALFF-LSAS**					
R Precentral gyms	F	249	33, -42, 63	3.58	0.001
**fALFF-LSAS**					
L Superior frontal gyrus	F	155	33, -42, 63	3.69	0.001
R Cerebellum anterior lobe	M	444	6, -45, -9	5.48	*p* < 0.001
L Superior temporal gyrus	M	198	-48, -12, -3	3.55	0.001
R Insula	M	173	45, -6, 9	3.41	0.001
R Postcentral gyms	F	154	-9, 36, 39	-3.76	*p* < 0.001
R Medial prefrontal gyrus	M	532	21, 39, -3	-6.28	*p* < 0.001
R Supramarginal gyms	M	101	54, -51, 30	-3.94	*p* < 0.001
R Precuneus	M	233	3, -69, 48	-3.73	*p* < 0.001


*CBSS, Cheek and Buss Shyness Scale; MNI, Montreal Neurological Institute; LSAS, Leibowitz Social Anxiety Scale; M, male; F, female.*

Significant correlation of LSAS with ALFF/fALFF maps in males: For the ALFF maps, no significant positive or negative correlation between the LSAS score and the ALFF maps was found. For the fALFF maps, there was a positive correlation with the LSAS scores in the left superior temporal gyrus, right cerebellum anterior lobe, and right insula in males as well as a negative correlation in the right medial frontal gyrus, right supramarginal gyrus, and right precuneus in males.

## Discussion

To the best of our knowledge, this is the first study to investigate sex-specific regional cerebral activity concerning shyness and social anxiety using ALFF/fALFF measurements through resting-state fMRI in young healthy adults. For shyness, we found a significant positive correlation with ALFF or fALFF in various cortical regions, including the fronto-parietal, temporal, and orbital gyrus and a negatively correlation in the bilateral insula, cerebellum anterior lobe, occipital, cingulate, and postcentral gyrus in females; in males, we only found a positive correlation in the left medial frontal gyrus and a negative correlation in the left superior parietal gyrus. For LSAS, a significant positive correlation was found in the frontal lobe and a negative correlation in the postcentral gyrus in females; however, more widespread functional impairments were found in males, including the left superior temporal gyrus, right cerebellum anterior lobe and right insula, right medial frontal gyrus, right supramarginal gyrus, and right precuneus. Thus, we demonstrate that shyness in healthy subjects is associated with the frontoparietal control network and cingulo-insular network, whereas social anxiety is associated with the frontal-limbic and frontoparietal control networks. Both of these patterns are influenced by sex. It also provides objective evidence for distinguishing shyness and social anxiety in terms of regional spontaneous brain activity.

Sex differences across human beings have been documented many times using different methods. Several studies, including a meta-analysis, have reported sex differences between males and females in terms of shyness, for example, higher rates of shyness in female children compared to male children (Else-Quest et al., 2006; Smith et al., 2012). In contrast, Engfer (1993) conducted a prospective study that examined the conditions that increased or decreased shyness, and their results showed that more girls (33%) than boys (less than 10%) ‘outgrew’ their shyness, suggesting that shyness is more stable in males than in females (Engfer, 1993). Reports of behavioral differences on shyness have been inconsistent, but some neuroimaging evidence suggests that males and females may engage different brain networks when processing outside stimuli. For example, a previous electroencephalography (EEG) study observed increased activation in shy females when processing emotional stimuli (Theall-Honey and Schmidt, 2006). In current study, we demonstrated that shy or social anxiety males and females may engage different brain networks in brain spontaneous activity using resting state fMRI. We found shyness was only positively related to brain spontaneous activity in the frontoparietal control network and negatively related to the cingulo-insular network in females but not males. Although the behavioral scores for shyness or social anxiety didn’t reach statistical difference in our study, the interesting thing is males had higher CBSS scores while female had higher LSAS scores. We postulate this may contribute to the fact that shy males and shy females engage different neural circuits and highlight the importance of considering sex in shyness research.

At the regional level, sex differences in the relationship between shyness and intrinsic brain activity in several brain regions were demonstrated. Specifically, for women, we found that shyness was positively related to brain spontaneous activity in the frontoparietal control network (mainly located in the dorsolateral prefrontal cortex, middle temporal motion complex, and anterior inferior parietal lobule), and negatively related to the cingulo-insular network (mainly located in the anterior cingulated cortex, insular and occipital cortex, and cerebellum). It is well known that both of these two networks are commonly implicated in cognitive control (Dosenbach et al., 2007; Vincent et al., 2008). However, these networks also show disparate functional properties: the frontoparietal control network is responsible for start cue-related activity and fit a proactive control pattern, whereas the cingulo-insular network responsible for reactive control pattern that affects downstream processing in a more stable fashion and performs switching between the default mode and task-related states of brain connectivity (Dosenbach et al., 2007; Braver et al., 2009). Braver et al. (2009) have recently developed a dual mechanisms of control model that distinction between proactive and reactive cognitive control. Proactive control, as a form of early selection, relies on the activity of the frontoparietal control network and reflects top–down regulation (Miller and Cohen, 2001; Braver et al., 2009). In contrast, reactive control was associated with activity of cingulo-insular network, which act as a “late correction” mechanism and reflects the active maintenance of task goals. For frontoparietal control network, this goal maintenance activity serves as a source of top–down bias that can facilitate processing of expected upcoming events that have a high cognitive demand. Similarly, higher social inhibition has also been found to be correlated with altered resting state connectivity in dorsal attention networks (Blackford et al., 2014). Consistent with this, shy individuals had showed enhanced brain activity in frontoparietal network when processing of imminent and ambiguous social threat (Tang et al., 2016). Given the function in frontoparietal network and related prior studies, the present study may provide evidence shy female will rely upon the anticipation and prevention of the interference in advance, and showed increased or effortful cognitive control. By comparison, the negative association with altered intrinsic function in the cingulo-insular network among shy females consistent with a reactive control profile, in which control is engaged only after a conflict is encountered and is driven by enhanced conflict monitoring activity.

Our previous study observed significant sex-by-shyness interactions in this network (Yang et al., 2015). In detail, the FCS values of these regions positively correlated with shyness scores in females but negatively correlated in males. Nonetheless, the FCS method can only allow us to identify higher strength of connectivity to other regions (i.e., an energy-efficient hub within a large network) (Yang et al., 2015) but cannot give us more detailed information for the activity pattern for the hub regions and connected regions (Bassett and Bullmore, 2009; Buckner et al., 2009). The present study revealed that shy females, but not males, showed decreased activity in the cingulo-insular network, perhaps reflecting decreased switching mechanisms in the neural networks, and lower of reactive control. Thus, overactivity in the frontoparietal network and hypoactivity in the cingulo-insular network in shy females may suggest that shy female may facilitate the use of the proactive control strategy other than reactive one to copy with outside social affairs.

To the contrary, for shy males, there is only a positive correlation in the left medial frontal gyrus and a negative correlation in the left superior parietal gyrus. Interestingly, the medial frontal gyrus appears to be situated somewhat inferior to the human homolog of the frontal eye fields and is also important for high-level executive functions and decision-related processes (Talati and Hirsch, 2005). The superior parietal gyrus is believed to play a major role in the top–down control of attention. The disequilibrium function in the parietal and frontal cortex may therefore implicate an impaired cognitive control in shy males. The gender differences observed in intrinsic brain activity, together with consistent findings across other studies, suggest widespread functional impairments in shy female individuals compared to males.

For social anxiety, our results revealed that the LSAS scores were positively associated with regional spontaneous activity in the frontal-limbic network and negatively associated in the frontal-parietal network in males, but less brain regions were involved in females, which differs from the brain activity pattern of shyness. Functional alterations of emotion-processing brain regions involving frontal-limbic circuitry are thought to be involved in the pathophysiology of anxiety disorders (Blair et al., 2008; Monk et al., 2008). Consistent with this, a previous study demonstrated that individuals who are at increased risk for anxiety disorder have increased activation in the frontal cortex and insula (Christensen et al., 2015). The insula are often highly associated with brain activity in the orbitofrontal cortex and superior temporal gyrus, and together they are purported to show mirroring properties, producing the constellation of anxiety-related symptoms that characterize impairments in reciprocal social interactions (Craig, 2005; Cattaneo and Rizzolatti, 2009; Nguyen et al., 2016). The higher activity of frontal-limbic region may therefore reflect a vulnerability for anxiety disorders that contributes to the development of anxiety symptoms. The negative association between ALFF/fALFF in the frontal-parietal network and LSAS is also found in our whole brain regression analyses. This finding is consistent with adult and pediatric fMRI studies that have also reported abnormalities of the frontoparietal network in anxiety disorders and high social anxiety individuals (Schmid et al., 2015). The predominant recruitment of frontoparietal network among participants with social anxiety is consistent with a proactive control profile, characterized by attentional focus and the early selection of an intended response strategy. The presence of functional alterations in the frontoparietal network in socially anxious individuals may reflect the impaired cognitive control abilities, especially relied additionally on a reactive control process in social anxiety.

More importantly, a pattern of hyperactivity in the frontal-limbic network and hypoactivity in the frontoparietal network is found in males but not in females. Interestingly, recent research conducted using a pot probe task found that males’ attentional bias to social threat was significantly positively correlated with their social anxiety, but no correlation was found in females (Zhao et al., 2014). The present study, consistent with the Zhao et al. (2014) study, fails to find hyperactivity of the limbic region in females. Despite sex differences in social anxiety disorder showing a preponderance of females over males of as much as 2 to 1 (Essau et al., 1999), our results seems to suggest that men have wider functional variations than women in social anxiety.

Another possible explanation for the neural functional differences in different genders discovered by current study is the culture issue. Previous study has found that shy girl was associated with positive outcome at both home and school whereas the opposite is true for boys (Chen et al., 1998, 2011). Similarly, high shy boys have significantly more internalizing problem than the high shy girls (Henderson et al., 1998). This difference may be attributed to the so called cultural expectations and socialization patterns referring to sex. For example, some degree of increased inhibition/shyness may be considered ‘gender appropriate’ in girls, but not in boys (Frick and Gresack, 2003; Dall, 2004).

It is notable that shyness is generally considered to be a normal personality trait, whereas social anxiety is viewed as a potential clinical disorder in the DSM-IV. Nevertheless, both of these conditions share many symptoms, including somatic, behavioral, and cognitive symptoms (Heiser et al., 2009), which raises the question about the diagnostic boundaries of social anxiety. In addition, research on the cerebral intrinsic function related to shy and socially anxiety has important clinical implications. Since previous study had proved that the brain network observed in the present study would change in activity after psychotherapy (Messina et al., 2016). As expected, we found specific intrinsic brain networks associated with shyness (or social anxiety): shyness was positively related to brain spontaneous activity in the frontoparietal control network and negatively related to the cingulo-insular network, whereas social anxiety was positively associated with regional spontaneous activity in the frontal-limbic network and negatively associated in the frontoparietal network. A possible explanation is that the presence of hyperactivity in the frontoparietal network and hypoactvity in the cingulo-insular network in shyness may reflect shyness engaged more proactive control process and decreased switching mechanisms. In contrast, the higher activity of the frontal-limbic region and hypoactivity in the frontal-parietal network in social anxiety perhaps indicates increased anxiety symptoms with lower proactive cognitive control ability in the socially anxious population. These results suggest that biological factors may contribute to determining the differences between shyness and social anxiety and lend support to the view that shyness should be considered as a distinct characteristic in terms of regional spontaneous activity.

Although the results of the fALFF and ALFF are generally similar in the correlation analysis of shyness and social anxiety, there are some discrepancies when applying different indexes in the present study. The altered spontaneous activity that was associated with shyness using ALFF was larger than the altered spontaneous activity using the fALFF approach. Previous studies have shown that the ALFF method is more sensitive to signal fluctuations contributed by physiological noise irrelevant to brain activity (Zou et al., 2008). Therefore, although we performed physiological noise removal using DPARSF, the results of the ALFF approach might still have been affected by these noises. The fALFF approach was used to overcome this disadvantage and has been shown to have improved sensitivity and specificity in the detection of spontaneous brain activity compared with the ALFF approach. Thus, the less altered brain activity related to shyness observed using the fALFF compared to the ALFF results was possibly caused by the effective suppression of the fALFF of the physiological signals. However, because the root mean square of the low-frequency oscillations in the white matter is approximately 60% lower than that in the gray matter, the ALFF measurement has higher test–retest reliability in gray matter than the fALFF measurement. This may help us to understand the more widespread altered brain regions associated with social anxiety when using fALFF. To avoid selection bias, we reported both the ALFF and fALFF indexes to investigate the strength of the neural oscillations associated with shyness and social anxiety, and these two indexes may provide complementary information about regional spontaneous brain activity.

Several additional issues need to be addressed. First, although we found a possible link between functional changes in a number of relevant brain areas that may underlie psychological aspects of shyness or social anxiety, the cross-sectional and resting-state design cannot establish direct causal roles. Second, several other factors are known to create sex differences between males and females, including hormones, menstruation, brain morphology, etc. Our study has not considered such factors regardless of the importance that they may possess. Future studies can elucidate how these individual factors may affect the sex differences in shyness and social anxiety by controlling for these factors one at a time and comparing the matched subjects in their studies. Furthermore, we chose normal subjects with different levels of shyness in our study. In future research, it would be interesting to compare males and females that could be characterized as bold subjects and extremely shy subjects, such as people with social anxiety disorder who tend to seek medical help.

## Conclusion

Our present study has shown that sex differences in intrinsic brain activity are related to shyness and social anxiety and also provides objective evidence for distinguishing shyness and social anxiety in terms of regional spontaneous brain activity in resting fMRI.

## Author Contributions

Conceived and designed the experiments: XqH, QG, and XY. Performed the experiments: XY, MZ, SL, LC, and XyH. Analyzed the data: SL, XY, MZ, TC, SW, and XqH. Contributed reagents/materials/analysis tools: YS and XqH. Wrote the paper: XY, MZ, SL, and XqH.

## Conflict of Interest Statement

The authors declare that the research was conducted in the absence of any commercial or financial relationships that could be construed as a potential conflict of interest.

## Footnotes

1 http://resting-fmri.sourceforge.net/

2 http://www.restfmri.net/
