# Supplementary material for: Intrinsic Brain Activity Responsible for Sex Differences in Shyness and Social Anxiety
Source: Front Behav Neurosci. 2017 Mar 13;11:43. doi: 10.3389/fnbeh.2017.00043 (PMC5346560; doi:10.3389/fnbeh.2017.00043)
Supplement: Supplementary file 1 [file Table_1.doc]

**Table S1.** Detailed information for voxels showing significant differences between males and females in rs-fMRI ALFF values with age and LSAS score as covariates with AlphaSim correction (cluster size > 22 mm^3^, p < 0.001)

| Gender | Region | Voxel Size | MNI Coordinates X,Y,Z | T value | *P* value |
| --- | --- | --- | --- | --- | --- |
| M>F | L Inferior Frontal Gyrus | 162 | -27,15,-12 | 4.67 | P<0.001 |
| M>F | R Inferior Frontal Gyrus | 30 | 36,30,-12 | 3.88 | P<0.001 |
| M>F | L Cerebellum Posterior Lobe | 25 | -6,-78,-24 | 3.72 | P<0.001 |
