# Supplementary material for: Intrinsic Brain Activity Responsible for Sex Differences in Shyness and Social Anxiety
Source: Front Behav Neurosci. 2017 Mar 13;11:43. doi: 10.3389/fnbeh.2017.00043 (PMC5346560; doi:10.3389/fnbeh.2017.00043)
Supplement: Supplementary file 2 [file Image_1.pdf]

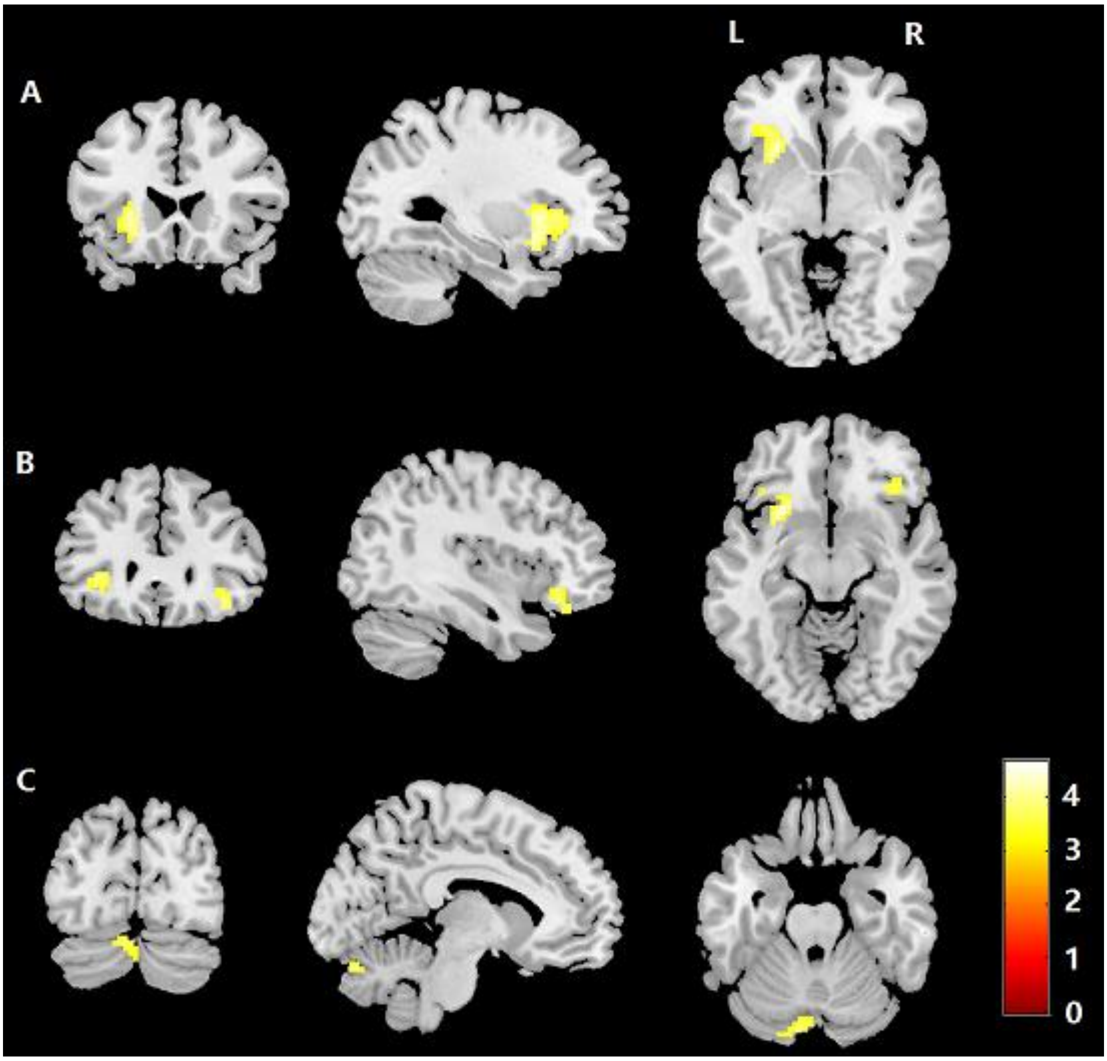

Fig S1. Regions showing significantly increased ALFF activity between males and females, with age and LSAS score as covariates. Yellow color indicates increased activity of the brain in males compared to females. A) Left Inferior Frontal Gyrus (LIFG), B) Right Inferior Frontal Gyrus (RIFG), C) Left Cerebellum Posterior Lobe (LCPL). The statistical threshold was set at p value <0.001, cluster size >22 mm^3 (AlphaSim corrected).
